# Cervical cancer stem cells manifest radioresistance: Association with upregulated AP-1 activity

**DOI:** 10.1038/s41598-017-05162-x

**Published:** 2017-07-06

**Authors:** Abhishek Tyagi, Kanchan Vishnoi, Harsimrut Kaur, Yogesh Srivastava, Bal Gangadhar Roy, Bhudev C. Das, Alok C. Bharti

**Affiliations:** 1 Molecular Oncology Laboratory, B.R. Ambedkar Centre for Biomedical Research (ACBR), University of Delhi, Delhi, 110007 India; 2 Division of Molecular Oncology, National Institute of Cancer Prevention and Research (NICPR), Noida, 201301 Uttar Pradesh India; 3 Stem Cell and Cancer Research Lab, Amity Institute of Molecular Medicine and Stem Cell Research (AIMMSCR), Amity University, Noida, Uttar Pradesh 201313 India; 4 Institute of Nuclear Medicine and Allied Sciences, Defence Research Development Organization, Delhi, 110 054 India; 5 Molecular Oncology Laboratory, Department of Zoology, University of Delhi, Delhi, 110007 India

## Abstract

Transcription factor AP-1 plays a central role in HPV-mediated cervical carcinogenesis. AP-1 has also been implicated in chemo-radio-resistance but the mechanism(s) remained unexplored. In the present study, cervical cancer stem-like cells (CaCxSLCs) isolated and enriched from cervical cancer cell lines SiHa and C33a demonstrated an elevated AP-1 DNA-binding activity in comparison to non-stem cervical cancer cells. Upon UV-irradiation, CaCxSLCs showed a UV exposure duration-dependent higher proliferation and highly increased AP-1 activity whereas it was completely abolished in non-stem cancer cells. CaCxSLCs also showed differential overexpression of c-Fos and c-Jun at transcript as well as in protein level. The loss of AP-1 activity and expression was accompanied by decrease in cell viability and proliferation in UV-irradiated non-stem cancer cells. Interestingly, CaCxSLCs treated with curcumin prior to UV-irradiation abolished AP-1 activity and a concomitant reduction in SP cells leading to abrogation of sphere forming ability, loss of proliferation, induction of apoptosis and the cells were poorly tumorigenic. The curcumin pre-treatment abolished the expression of c-Fos and c-Jun but upregulated Fra-1 expression in UV-irradiated CaCxSLCs. Thus, the study suggests a critical role of AP-1 protein in the manifestation of radioresistance but targeting with curcumin helps in radiosensitizing CaCxSLCs through upregulation of Fra-1.

## Introduction

Cervical cancer is a major reproductive health problem in women of developing countries^1^. Despite it being a preventable cancer with a long precancerous stage, most women from resource poor countries do not have access to effective screening or vaccination program and are often detected in an advanced stage of cancers. When treated, these cancers often develop chemo-radioresistance leading to treatment failure, loco-regional recurrences or distant metastasis^2^. Although the 5-year overall survival rate of advanced stage cervical cancer has improved with chemo-radiotherapy, tumors with similar pathological grade and stage, are often not equally sensitive to radiation. Emerging data suggest presence of a hierarchically-organized small population of cancer stem cells (CSCs) that are inherently resistant to radiation and other anti-cancer drugs^3^ and variable degree of their presence/activity may determine the extent of the chemo-radioresistance^4^. The CSCs derived from primary cervical tumors^5, 6^ and cervical cancer cell lines^6–8^ demonstrated an increased radioresistance^7, 9^. However, the mechanism(s) responsible for manifestation of radioresistance by these CSCs are poorly understood.

Studies carried out from our group demonstrated a pivotal role of aberrantly expressed and constitutively active transcription factor AP-1 that increased with the severity of lesions during cervical carcinogenesis^10^. AP-1 has also been demonstrated as a key regulator for expression of HPV oncogenes E6 and E7^10–13^. Studies also showed a critical role of AP-1 in mediating chemo- and/or radioresistance^14, 15^. AP-1 activity is constituted by homo-dimerization of members of Jun (c-Jun, JunB, JunD) or their hetero-dimerization of the members of Fos (cFos, FosB, Fra1, Fra2) family proteins^16^. Subsequent studies showed involvement of AP-1 family members in mediating resistance to anti-cancer therapies demonstrated a potential role of c-Jun in chemo-radioresistance in head and neck cancers^17^. Recently, our group also showed pivotal role of c-Jun and Fra-2/c-Fos in aggressive tumorigenesis, metastasis and chemo radioresistance of tongue cancer^18^. Further studies demonstrated c-Jun phosphorylation through HIF-1α upregulate Beclin-1 mRNA and protein expression that contributed to radioresistance in lung cancer^19^. Similarly, RNAi-mediated knockdown of the c-Jun gene sensitized human nasopharyngeal carcinoma cells to radiation^20^. On the other hand, Fra-1, Fra-2 and JunD were shown to contribute to prostate cancer growth and survival after radiation. Increased expression of phosphorylated c-Jun and c-Fos were found essential for induction of apoptosis in response to UV irradiation^21^ indicating cell to cell context dependent c-Jun functional variation. Nevertheless, these studies along with investigations involving inhibition of AP-1 activity^14, 21^ reemphasized important role of AP-1 in governing radioresistance but the effect were AP-1 member-specific in different cancers. Recent investigations suggest that AP-1 could play a pivotal role in governing radio- or chemo-resistance of CSCs^21, 22^. But it is not understood how AP-1 which governs oncogenic activity of HPV is involved in manifestation of radioresistance of CSCs in cervical cancer. Curcumin, a pharmacologically safe herbal compound is a potent inhibitor of AP-1 and HPV in cervical and oral cancer cells^10, 23^. Curcumin has been shown to act as a radiosensitizer^24, 25^ but the mechanism by which curcumin alleviate radioresistance is not clear.

We report here the mechanistic role of AP-1 in survival and radioresistance of cervical CSCs and demonstrate therapeutic utility of curcumin as an AP-1 inhibitor that may serve as an adjuvant to make chemo-radiotherapy most effective by sensitizing the cancer and cancer stem cells.

## Results

### Side population cells represent a distinct group of putative cervical cancer stem-like cells: a functional and molecular characterization

**Figure 1:**
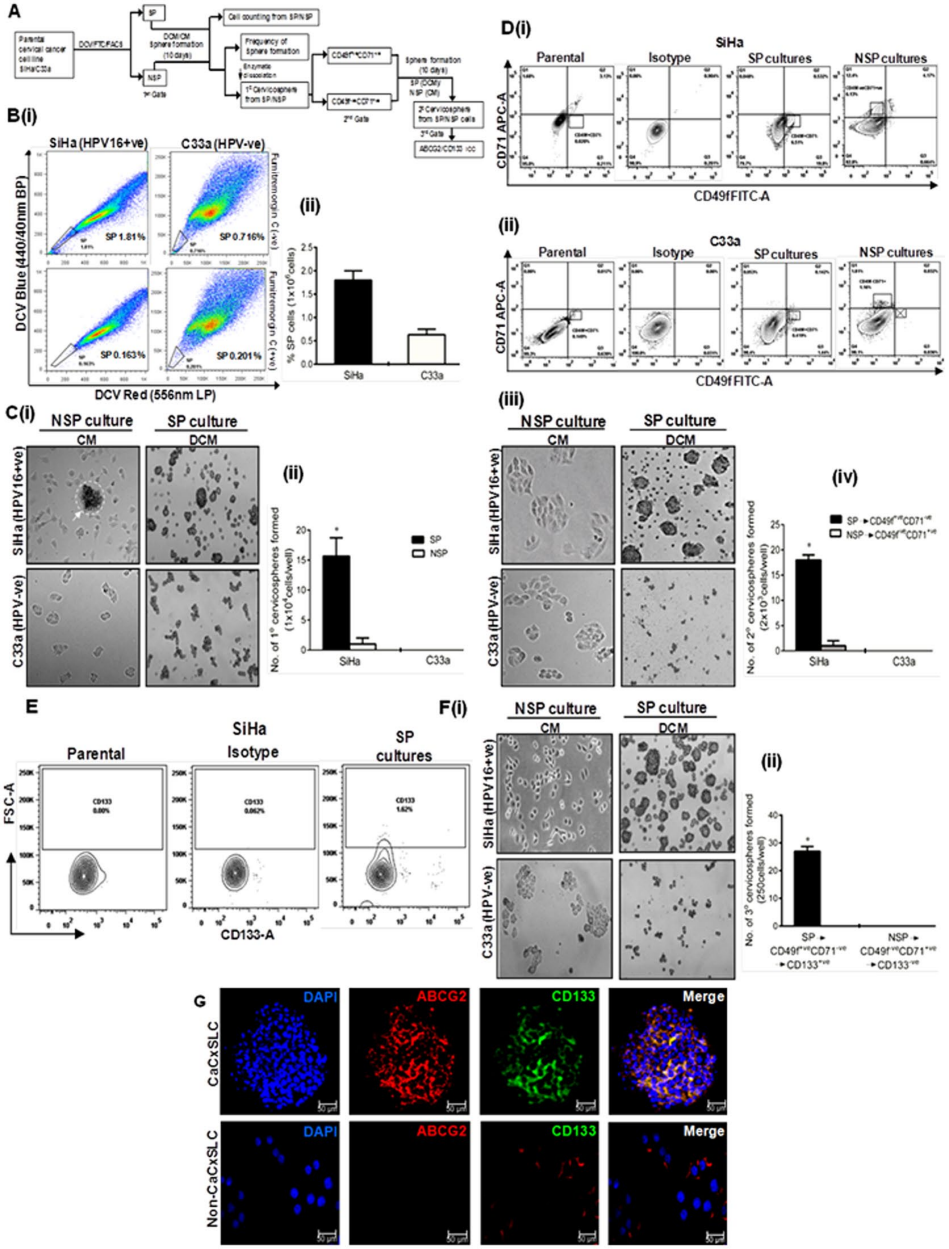
Isolation and enrichment of cervical cancer stem-like cells. (**A**) Schematic representation of the experimental design showing sequential gating. (**B**) Flowcytometric analysis of DCV-stained cervical cancer cells showing FTC-sensitive, DCV low cells designated as SP cells. (i) Representative dot-plot and (ii) cumulative data on three independent experiments showing proportion (mean ± SD) of cells in SP region. (**C**) Cervicosphere formation in day10 cultures seeded with sorted SP and non-SP cells in low adherence defined conditioned medium (DCM) and complete medium (CM) respectively (original magnification-100x) (i). Cumulative 1°cervicosphere frequency (mean ± SD) in 10 days culture of SP and non-SP cells (seeding density of 10,000 cells/well) in three independent experiments (ii). *p-value < 0.05 vs. NSP cultures. (**D**) Flowcytometric analysis of cells derived from enzymatically-dissociated primary cervicospheres from SP cultures (DCM) or adherent monolayer derived from NSP cultures of SiHa (i) and C33a cells (ii) for dual labelling of CD49f and CD71 cell surface marker to check 2°cervicosphere formation for 10 days. (iii) Frequencies (mean ± SD) of 2°cervicospheres in cultures seeded with specific cells from CD49f^+ve^CD71^−ve^ quadrant from SP culture and CD49f^−ve^ CD71^+ve^ quadrant from NSP culture in DCM or CM respectively on low adherence environment. Cells seeded with a density of 2000 cells/well in three independent experiments. *p-value < 0.05 vs. NSP cultures. (**E**) CD133 expression in cells from 2°cervicospheres derived from SP → (CD49f^+ve^CD71^−ve^) or cells from parent cultures (SiHa). (**F**)(i) 3°cervicospheres from sequential triple gating SP → (CD49f^+ve^CD71^−ve^) → CD133^+ve^ at day10 cultures (orginal magnification–100x). (ii) Frequencies of 3°cervicospheres at day10 formed in three independent experiments. Seeding density (250 cells/well). *p-value < 0.05 vs. control NSP cultures. (**G**) Expression of ABCG2 and CD133 in 3°cervicospheres at day10 generated from [SP → (CD49f^+ve^CD71^−ve^) → CD133^+ve^] designated as CaCxSLCs and from [NSP → (CD49f^−ve^ CD71^+ve^) → CD133^−ve^] designated as non-CaCxSLC cultures.

Cervical cancer stem cells were isolated from HPV-positive and HPV-negative cervical cancer cell lines by triple gating as described in Fig. 1A. The process included isolation of side population (SP) cells followed by culturing and regated on phenotypic markers CD49f and CD71 and then finally gating on CD133 (Fig. 1B–E). The sorted cells were examined for their stemness property by cervicosphere formation assay using intermittent culturing in low adherence defined conditioned medium (DCM). Sorted cells subjected to sphere formation generated cervicospheres only in cultures seeded with SP → (CD49f^+ve^ CD71^−ve^) → CD133^+ve^ cells which were designated as CaCxSLCs. Cervicospheres were absent in non-side population (NSP) [NSP → (CD49f^−ve^CD71^+ve^) → CD133^−ve^] (Fig. 1F) designated as non-CaCxSLCs when they were cultured on comparable media and surface condition as it resulted in anoikis of cells as reported earlier^26^. Therefore to use these sorted NSP cells as reference, alternate surface and condition were standardised using adherent plates coated with LMP-agarose in complete media (CM) and were used as reference (see Supplementary Fig. S1) as described earlier^6^. Confocal microscopy analysis of cervicospheres derived from CaCxSLCs and non-CaCxSLCs cultures revealed CD133 and ABCG2 expression in peripheral cells of the cervicosphere and these punctate markers were found to co-localize (Fig. 1G). In contrast, non-CaCxSLCs (control) showed a mild positivity for only ABCG2 whereas CD133 expression was completely absent.

### Constitutive activation of AP-1 in CaCxSLCs before and after UV-irradiation

**Figure 2:**
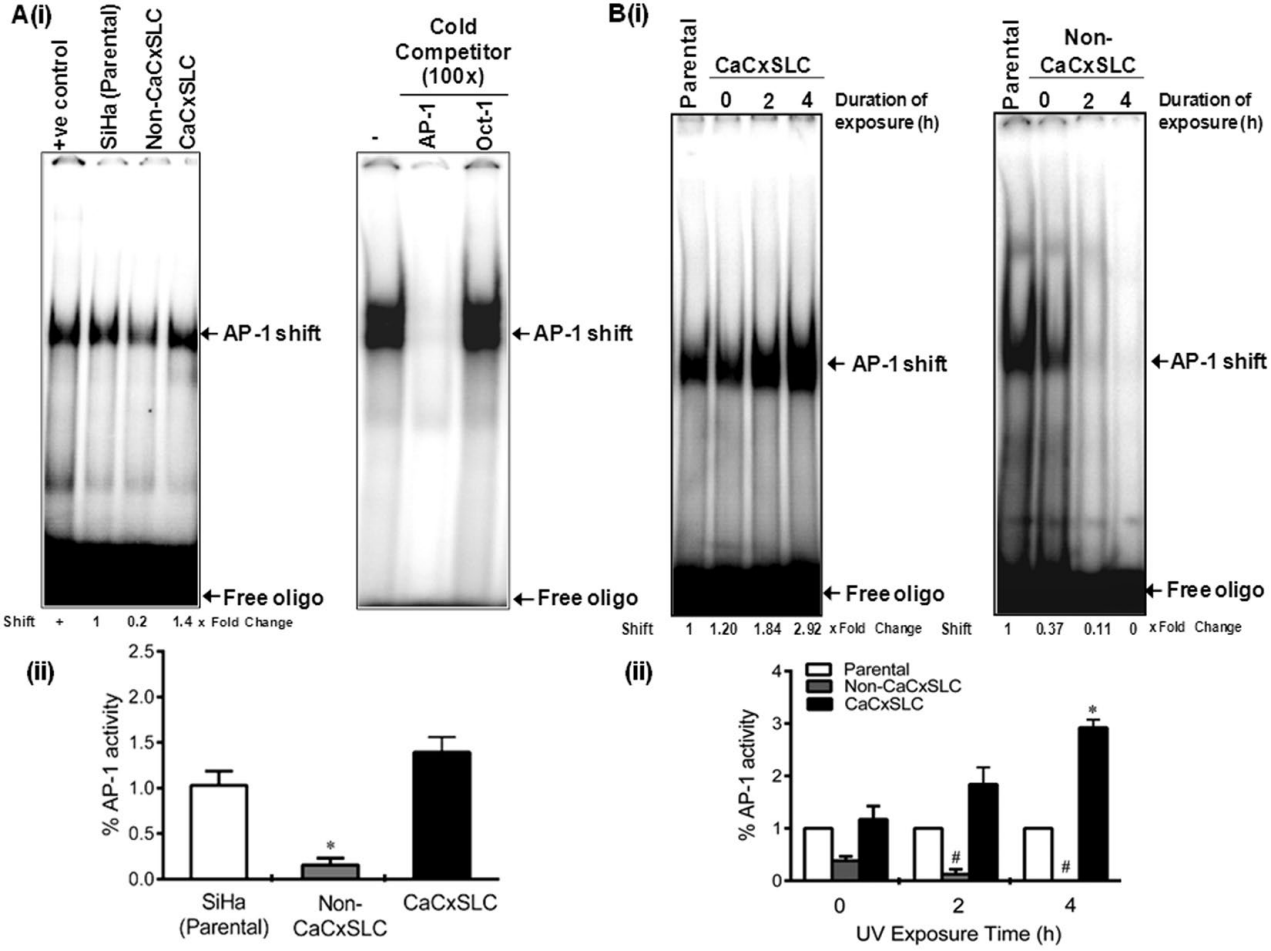
Effect of UVC-treatment on endogenous AP-1 activity and cervicosphere formation in CaCxSLC. (**A**) Status of AP-1 signaling in CaCxSLCs derived from SiHa cells. Representative autoradiogram showing AP-1 specific DNA-binding activity in cells from parental, cervicospheres derived CaCxSLCs and non-CaCxSLCs. Nuclear protein (10 µg/lane) from CaCxSLC cells (DCM) or non-CaCxSLCs cells (CM) were co-incubated with radiolabelled (γ-^32^P-ATP) AP-1 consensus sequence that contain AP-1 binding sequence and AP-1 specific DNA-binding was examined (i). AP-1 specific DNA-binding activity was verified by cold competition as described in Method by co-incubating 100x molar excess of either unlabelled homologous AP-1 probe or heterologous Oct-1 probe (ii). Numbers on the bottom represent fold change in AP-1 binding vs. parental cells. Data are expressed as the mean ± SD of three independent experiments (iii). *p-value < 0.05 vs. untreated controls i.e. CaCxSLCs and non-CaCxSLC cells. Untreated parental SiHa cells were included as positive control. (**B**) Effect of UV on AP-1 DNA-binding activity. Representative autoradiogram showing AP-1 DNA-binding in nuclear proteins (10 µg/lane) derived from cells of indicated culture that were exposed to 100 J/m^2^ UV radiation for variable time interval and harvested after 12 h after completion of the treatment (i). Cumulative quantitative densitometry data of three independent experiments (ii). *p-value < 0.05 vs. untreated cervicospheres of CaCxSLCs, ^#^ *p-value* < 0.05 vs untreated non-CaCxSLCs.

We examined the status of AP-1 binding activity in cells from CaCxSLC-derived cervicospheres in comparison to corresponding non-CaCxSLCs or parental SiHa cells with or without UV irradiation. A strong AP-1 specific binding activity was observed in CaCxSLCs in contrast to non-CaCxSLCs or parental SiHa cells (Fig. 2A). Interestingly, the cultures containing cervicospheres/CaCxSLCs and non-CaCxSLCs behaved differentially to UV (100 J/m^2^) radiation in terms of their response of AP-1 specific DNA binding activity. A characteristic increase in cellular AP-1 activity was observed in CaCxSLCs that increased with increase in the duration of UV exposure. In contrast, non-CaCxSLCs cells completely lost their AP-1 activity in cultures exposed to UV within 2 h (Fig. 2B).

### Elevated expression of AP-1 family proteins in CaCxSLCs

**Figure 3:**
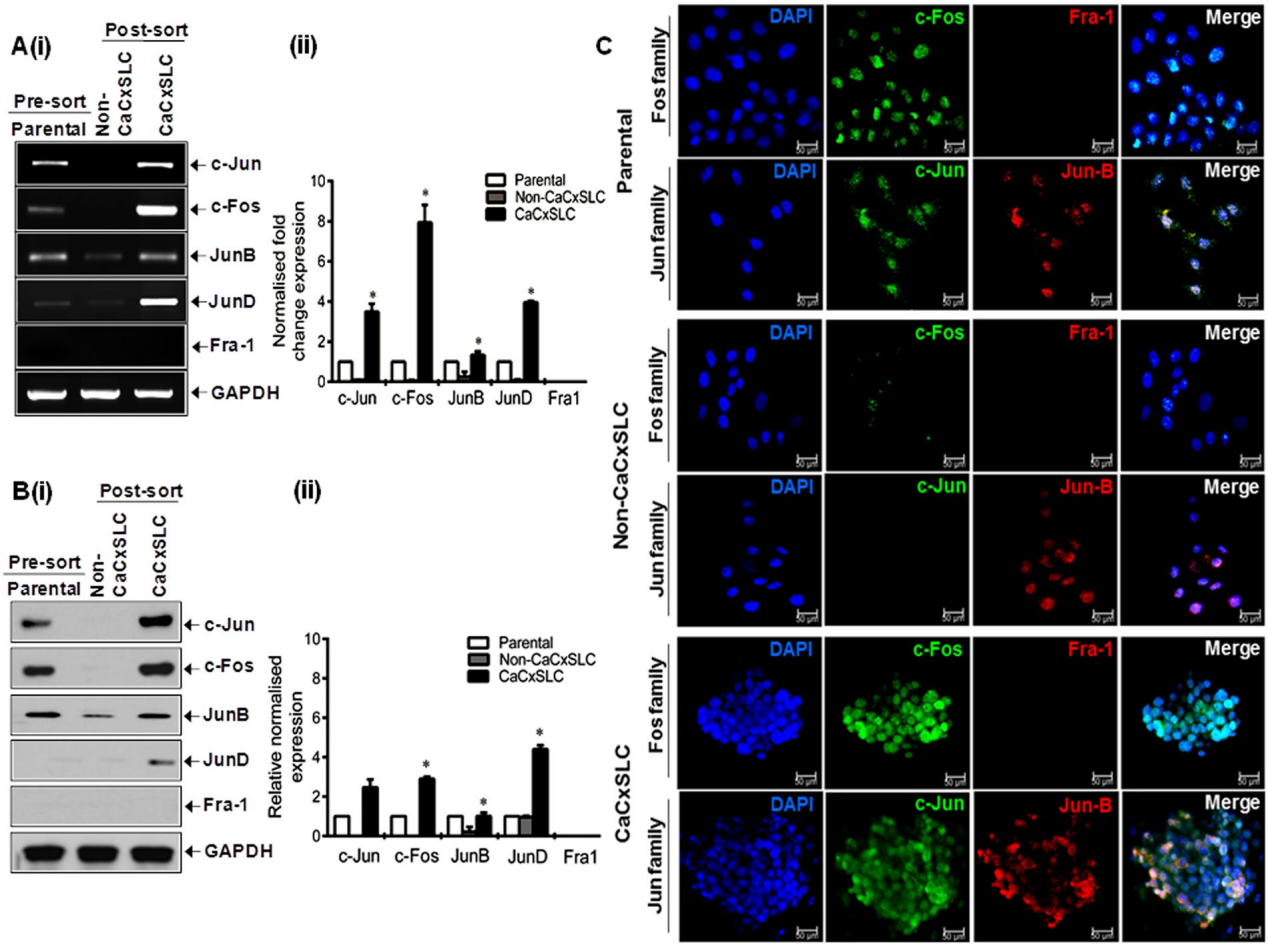
Expression of AP-1 members in CaCxSLCs. (**A**) Transcript levels of AP-1 family members. Representative cropped gel photographs showing relative transcript levels of indicated AP-1 members in cDNA prepared from CaCxSLCs and non-CaCxSLCs by qRT-PCR. cDNA from parental SiHa cells were used as reference. GAPDH qRT-PCR was used as input control for normalization as described in Methods (i). The gels were run under the same experimental conditions. Normalized fold change in transcript levels is expressed as mean ± SD of three independent experiments (ii). *p-value < 0.05 vs. parental SiHa cells. (**B**) Representative cropped blots showing expression levels of c-Jun, c-Fos, JunB, JunD and Fra-1 in total cellular extracts derived from CaCxSLCs culture and non-CaCxSLCs or presorted SiHa cultures. β-actin was used as input control (i). The abundance ratio of each AP-1 members to β-actin was analyzed by densitometry. The gels were run under the same experimental conditions. The relative normalized fold change in the protein is expressed as the mean ± SD of three independent experiments (ii). *p-value < 0.05 vs. parental SiHa cells. (**C**) Intracellular expression of cellular AP-1 protein in CaCxSLCs. Representative confocal immunofluuorescence image of AP-1 family members in day10 cultured cervicosphere CaCxSLCs and non-CaCxSLCs cultures. Parental SiHa cells were used as reference.

Assessment of the basal expression pattern of AP-1 family members in cells from CaCxSLC-derived cervicospheres by qRT-PCR analysis revealed elevated levels of major AP-1 family members (c-Jun, c-Fos, JunB and JunD) in CaCxSLCs as compared to parental or non-CaCxSLC cells (Fig. 3A). These cells, however, lacked Fra-1 transcript. Alteration in expression of AP-1 family members were further evaluated at protein level by immunoblotting that corroborated elevated expression of AP-1 members (c-Jun, c-Fos and JunB) (Fig. 3B). *In situ* analysis of AP-1 proteins by confocal microscopy in CaCxSLC cells in cervicospheres and non-CaCxSLCs or parental cells in respective cultures by intracellular immunofluorescence staining demonstrated a strong presence of c-Jun and c-Fos protein in CaCxSLCs and they were localised to nuclei of CaCxSLCs in cervicospheres (Fig. 3C). In addition, some of the CaCxSLCs showed concomitant expression of JunB and JunD. On the contrary, the non-CaCxSLCs cells showed minor expression of JunB.

### UV-irradiated cervicospheres display upregulated expression of c-Fos and c-Jun

**Figure 4:**
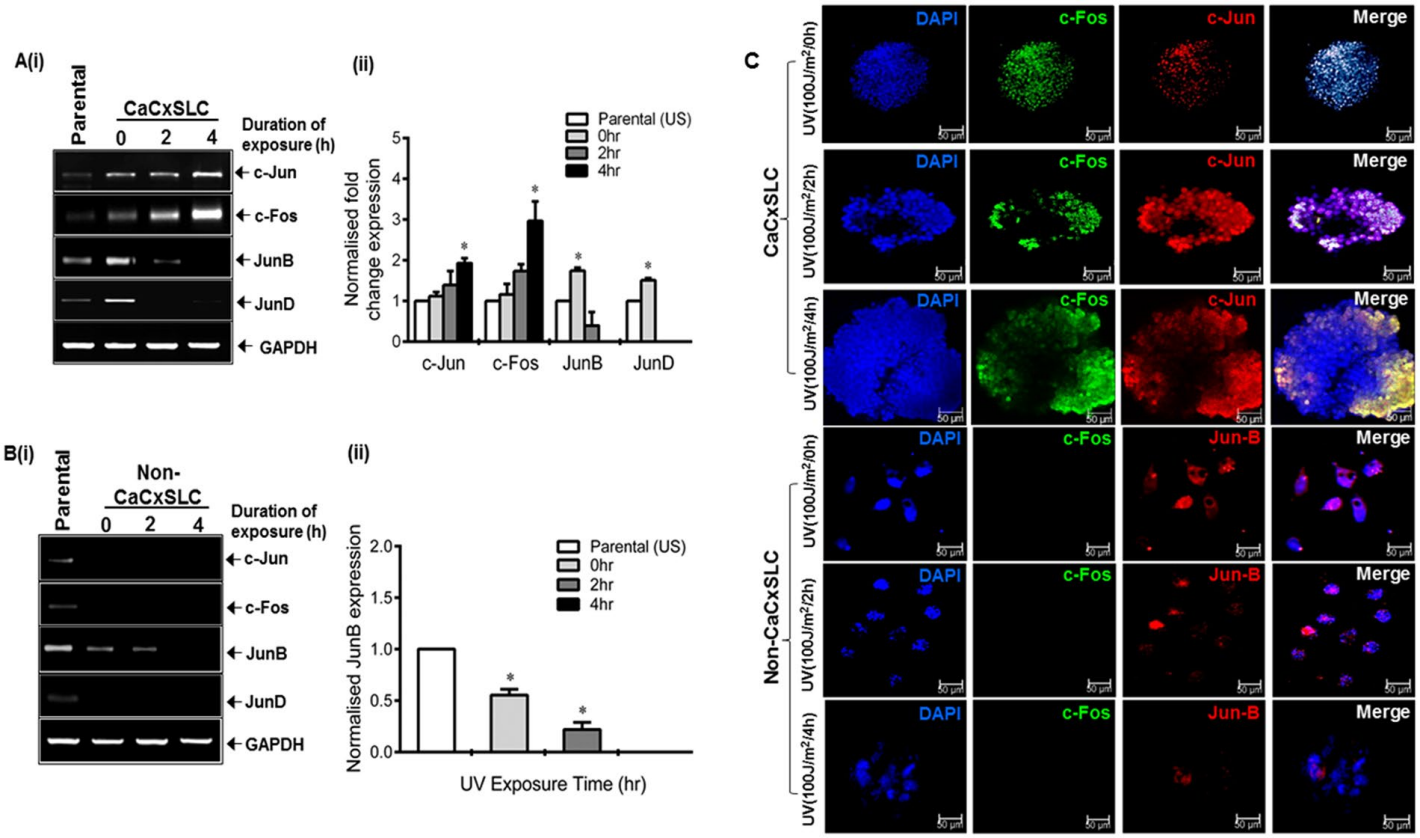
Effect of UV on cervicosphere and expression and localization of AP-1 proteins. (**A**,**B**) Effect of UV irradiation on transcript levels of respective AP-1 family members. Representative cropped gel photograph showing specific transcript levels of c-Jun, Jun-D, c-Fos and Fra-1 in cDNA prepared from untreated CaCxSLCs and corresponding non-CaCxSLC cells by qRT-PCR (**A**(i), **B**(i)). Parental SiHa (unsorted) cells were used as reference. GAPDH qRT-PCR was used as input control for normalization as described in Methods. The gels were run under the same experimental conditions. Normalized fold change in transcript levels is expressed as mean ± SD of three independent experiments (**A**(ii), **B**(ii)). *p-value < 0.05 vs. parental SiHa cells. (**C**) Representative confocal immunofluorescence image of 10 day CaCxSLCs or non-CaCxSLCs cultures treated with indicated exposure to UVC radiation. Each culture were fixed and stained with primary (c-Fos, c-Jun or Jun-B) and secondary antibodies [Alexa-488 (Green) conjugated goat anti-mouse or Alexa-594 (Red) conjugated goat anti-rabbit] and counterstained with DAPI (Blue) to visualize nuclei. White spots in merged photograph indicate co-localization of AP-1 in nuclei.

Considering an active and persistent AP-1 signaling operating in CaCxSLCs in UV-irradiated cervicospheres, these cells were examined for expression of various AP-1 members in cells from CaCxSLC-derived cervicospheres and cells of non-CaCxSLCs monolayers. cDNA prepared from control and treated cultures demonstrated a specific increase particularly in the level of c-Jun and c-Fos transcripts whereas JunD level showed a sharp decline in CaCxSLCs (Fig. 4A). In contrast, non-CaCxSLC cells showed complete abolition of JunB transcript level within 4 hr of UV exposure (Fig. 4B). Further examination by confocal microscopy revealed conspicuously increased expression and nuclear localization of c-Fos and c-Jun in CaCxSLCs. On other hand, non-CaCxSLCs cells lost their JunB levels with UV exposure that corresponded with the transcript levels of JunB (Fig. 4C).

### Downregulation of AP-1 activity by curcumin results in elimination of SP population and upregulation of Fra-1

**Figure 5:**
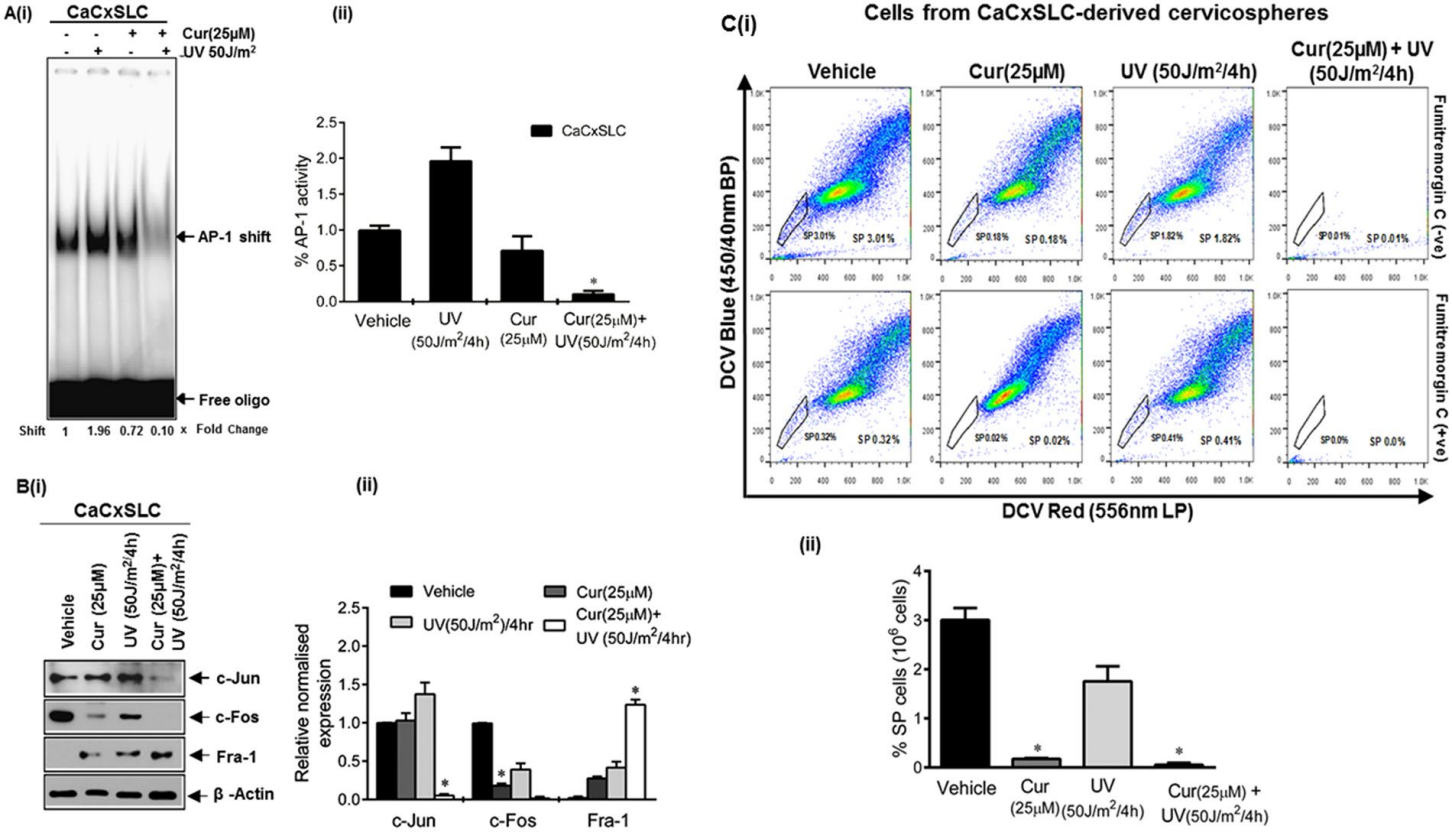
Effect of blocking AP-1 activity and expression by curcumin on SP cells. (**A**) Effect on AP-1 DNA-binding activity. Representative autoradiogram of nuclear protein (10 µg/lane) from day10 cervicospheres cultures treated with curcumin and/or UV co-incubated with labelled AP-1 probe and run on 5% native PAGE as described in Methods (i). Data are expressed as the mean ± SD of three independent experiments (ii). *p-value < 0.05 vs. vehicle treated cells from CaCxSLC-derived cervicospheres. (**B**) Immunoblot analysis of treated cells as described above. Representative cropped blots showing expression levels of AP-1 family members in day10 cervicospheres cells post-treatment of vehicle or cucurmin or UV or both. β-actin was used as input control (i). The gels were run under the same experimental conditions. Data are expressed as the mean ± SD of three independent experiments (ii). *p-value < 0.05 vs. vehicle treated cells from CaCxSLC-derived cervicospheres. (**C**) Curcumin eliminates SP cells post UV-irradiation. Dot-plot showing flowcytometric analysis of enzymatically dissociated and pooled cells from CaCxSLC-derived cervicospheres stained with DCV and/or FTC and incubated further with vehicle or curcumin alone for 24 h and subjected to UV radiation for 4 hr and cultures were left for another 12 hr for assessment of % SP cells in 10^6^ cells (i). Data are expressed as the mean ± SD of three independent experiments (ii). *p-value < 0.05 vs. vehicle treated cervicospheres cells.

Since increased AP-1 binding activity and expression was found to contribute to radioresistance manifested by CaCxSLCs, we assessed the effect of curcumin, on AP-1 activity by pretreating cervicospheres before UV exposure (50 J/m^2^). EMSA performed for AP-1 specific DNA binding activity on the nuclear proteins isolated from curcumin and UV-exposed cervicospheres along with their respective untreated controls demonstrated UV exposure duration dependent inhibition of AP-1 specific binding in CaCxSLC cells of curcumin-treated cervicospheres. The AP-1 activity was completely abolished when curcumin-pretreated CaCxSLCs were subjected to UV-radiation (Fig. 5A). CaCxSLCs derived cells from UV-treated cervicospheres showed almost complete downregulation of c-Fos and c-Jun but upregulation of Fra-1 (Fig. 5B). The SP analysis of cells from CaCxSLC derived vehicle treated cervicospheres demonstrated a characteristically higher percentage of SP cells (3.01% vs. 1.81% in parental cultures), which represent tumorigenic and resistant cells. Upon curcumin treatment, these cervicosphere cultures showed a specific decline in SP cell population (>90%) while UV-treated cervicospheres showed only a marginal change in SP population (30–40%). On the contrary, cells pretreated with curcumin and subjected to UV irradiation showed nearly complete elimination of all the cells (Fig. 5C). Therefore, the combinatorial treatment was capable of killing both, SP and the non-SP cells, in CaCxSLCs-derived cervicospheres.

### Combinatorial interaction of curcumin and UV-irradiation inhibit cell proliferation and induce apoptosis

**Figure 6:**
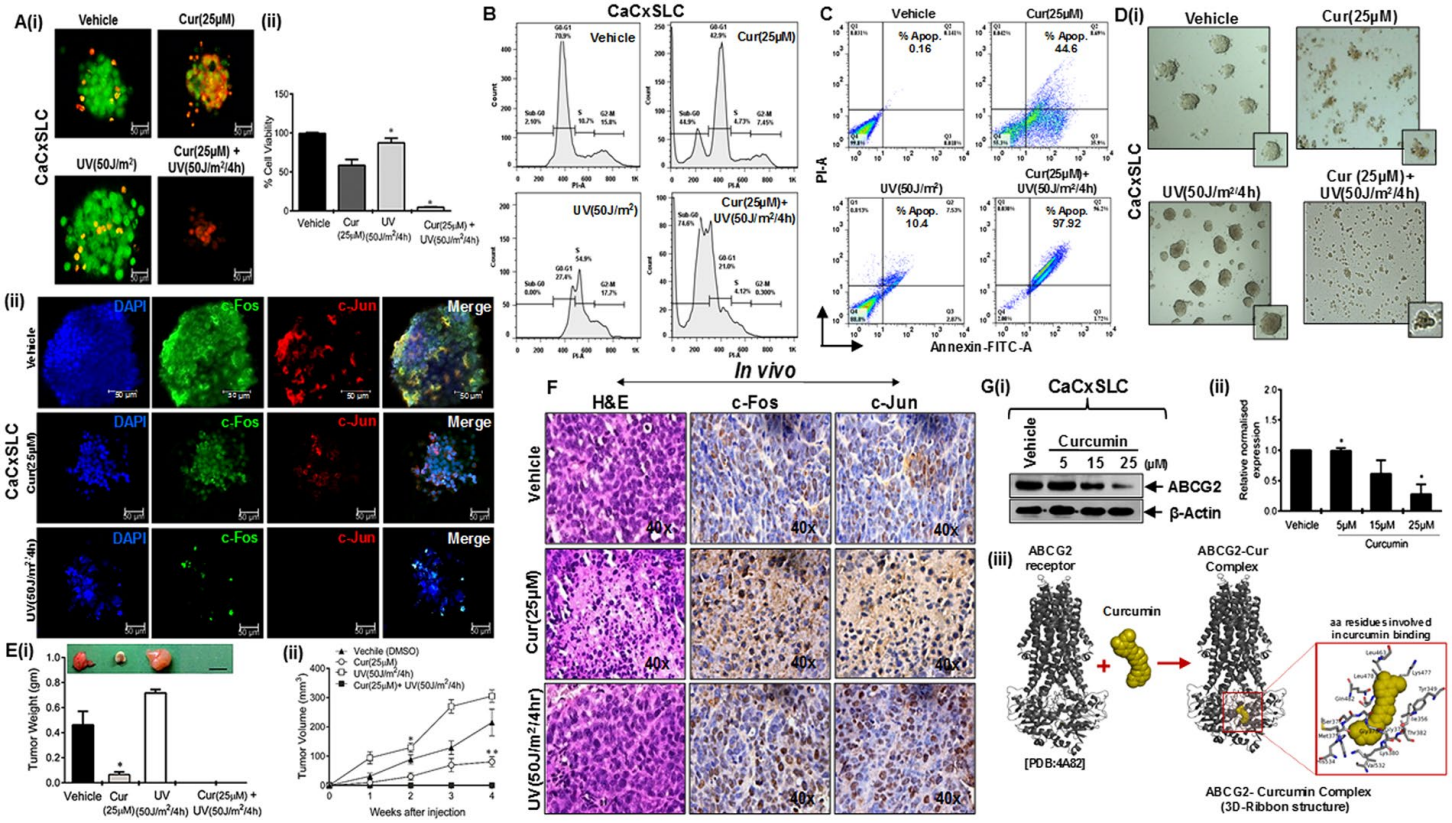
Combinatorial interaction of curcumin and UV-radiation on cell proliferation and survival. (**A**) Effect of curcumin and UV-irradiation on cell proliferation. Representative immunofluorescence image of day10 cervicospheres derived from CaCxSLCs treated with curcumin(24 hr) or UV-irradiation(4 hr) or in-combination by acridine orange/ethidium bromide (AO/EtBr) staining as described in Methods. Scale bar = 50 μm. (**B**) Effect of curcumin and UV-irradiation on cell cycle. Representative histogram showing cell cycle distribution as assessed by propidium iodide (PI) staining. (**C**) Flowcytometric analysis of day10 cervicospheres derived from CaCxSLCs treated as mentioned above and analyzed using AnnexinV-FITC apoptosis detection kit as per manufacturer’s instructions described in Methods. (**D**) Assessment of cervicosphere forming ability. Representative photomicrograph of day10 cervicospheres derived from treated CaCxSLCs under low adherence environment in DCM (original magnification-100x) (i). Representative confocal immunofluorescence image of day10 CaCxSLC cultures treated with curcumin(24 hr) and/or UV-radiation(4 hr). Each cultures were fixed and stained with primary (c-Fos and c-Jun) and (Alexa-488 or Alexa-594 conjugated goat anti-mouse or anti-rabbit) secondary antibodies and counterstained with DAPI. Merged photograph indicate co-localization of AP-1 in nuclei. (**E**) Tumor weight derived from post-treated CaCxSLCs (20 × 10^3^) measured after 4-weeks post-injection in the athymic nude mice (i). Data presented as mean ± SE, *p value < 0.05 w.r.t. vehicle treated tumor. Tumor growth curves of post-treated CaCxSLCs (20 × 10^3^ each) at 4-weeks post-injection in the athymic nude mice (ii). Data presented as the mean ± SE, *p value < 0.05 w.r.t. vehicle treated tumor. (**F**) H&E staining and IHC of tumors derived from curcumin or UV treated CaCxSLCs (original magnification-400x). (**G**) Representative cropped blots showing expression level of ABCG2 in extracts prepared from CaCxSLCs treated with different doses of curcumin(24 hr). β-actin was used as input control (i). The gels were run under the same experimental conditions. The abundance ratio of ABCG2 to β-actin was analyzed by densitometry. Data are expressed as the mean ± SD of three independent experiments (ii). *p-value < 0.05 vs. vehicle treated cells from CaCxSLC-derived cervicospheres. (iii). Representative 3D-ribbon structure of ABCG2(grey) and curcumin(yellow) complex. Red square showing binding area of curcumin in ABCG2 receptor. Close-up view showing ABCG2 amino-acid residues involved with curcumin binding.

To validate the above findings, we assessed the combinatorial effect of curcumin and UV-irradiated cultures for cell proliferation and apoptosis. Cervicospheres from CaCxSLCs derived cultures showed increased proliferation when subjected to UV radiation while curcumin pretreated UV-irradiated cervicosphere cultures showed complete disruption of cervicosphere as compared to curcumin alone (Fig. 6A). Moreover, flowcytometry-based cell cycle analysis of these cultures subjected to UV-radiation demonstrated majority of cells in S-phase in cultures as compared to cultures subjected to curcumin alone or in combination where majority of cells were in sub-G0 phase of cell cycle (Fig. 6B). These observations were further validated by staining with Annexin/PI that showed induction of apoptosis in curcumin treated cervicospheres which further increased when cultures were treated in combination with curcumin and UV-radiation (Fig. 6C). On the contrary, UV-treated culture showed only a small proportion of cells undergoing apoptotic cell death. To further dissect the combinatorial effect of curcumin and UV-radiation, treated CaCxSLCs were re-examined in cervicosphere cultures that showed complete inhibition of sphere formation in curcumin-treated UV exposed cultures (Fig. 6D). Confocal microscopy of elevated expression of AP-1 family members in cervicospheres from CaCxSLCs treated with curcumin or UV-radiation also showed downregulation of c-Fos and c-Jun (Fig. 6D). *In vivo* assessment of CaCxSLCs cells from cervicospheres treated with combination of curcumin and UV in nude mice demonstrated absence of tumor growth following s.c. injection of cells in nude mice (Fig. 6E). On the contrary, CaCxSLC cells treated with curcumin alone display delayed or retarded tumor growth as compared to untreated cells. Interestingly, cells from cervicospheres exposed to UV-radiation exhibited enhanced tumorigenicity and faster tumor growth (Fig. 6E). Histopathological analysis of tumors generated from curcumin injected CaCxSLC cells showed morphological features of apoptosis as compared to UV-radiation generated tumors (Fig. 6F). Immunohistochemical staining with anti-cFos and anti-cJun further confirmed above observation and showed enhanced positive staining of cFos and cJun in vehicle or UV derived tumors as compared to those in curcumin pretreated tumors which showed apoptotic cell death within the tumor milieu with low expression of c-Fos and c-Jun (Fig. 6F). Immunoblotting using ABCG2 antibody demonstrated lower expression of ABCG2 in CaCxSLC cultures treated with different concentration of curcumin in a dose-dependent manner (Fig. 6G). *In silico* docking analysis of curcumin on ABCG2 revealed complex formation of curcumin with ABCG2 receptor (Fig. 6G).

## Discussion

A small subset of stem cell population (CaCxSLCs) isolated from HPV16 positive cervical cancer cell line was employed to examine the role of transcription factor AP-1 following exposure of cells to UV radiation. Though cervical cancer cells possess a constitutively active AP-1^10^, the CaCxSLCs showed a much higher AP-1 binding as compared to non-CaCxSLCs or unsorted parental cells. Interestingly, the response of the two cell types to UV radiation varied significantly. CaCxSLCs showed an increased AP-1 activity, while it was completely abolished in the non-CaCxSLCs. The expression of AP-1 family proteins c-Jun, c-Fos, JunB and JunD also differed significantly as they were overexpressed in CaCxSLCs but their expression was low or undetectable in non-CaCxSLCs. Interestingly, level of c-Jun and c-Fos expression in CaCxSLCs increased as a function of duration and dose of UV radiation whereas JunB and JunD showed a significantly decreased expression. A similar decline in JunB was also observed in non-CaCxSLCs. These alterations in AP-1 expression and activity were translated in a strong increase in the cervicosphere size in CaCxSLC cultures and a corresponding loss of cell viability in non-CaCxSLCs. Pre-treatment of CaCxSLC cultures with curcumin prior to UV exposure not only abrogated the basal AP-1 activity but also prevented post-exposure UV-induced increase in constitutively active AP-1 in these cells. Further analysis of AP-1 members demonstrated that curcumin-treated cells displayed a significant decline in c-Jun and c-Fos level. Loss of AP-1 activity was accompanied by loss of CaCxSLCs post-UV exposure and resulted in collapse of tumorigenic cervicospheres by inducing apoptosis in these sphere derived cells. In an *in vivo* set-up, cervicosphere cells treated with UV showed a higher tumorigenic potential in tumor xenograft model in nude mice indicative of radioresistance. On the other hand, if the cells were pretreated with curcumin prior to UV irradiation, the tumors completely failed to form thus showing a potential involvement of constitutively active AP-1 in tumor-initiation and radioresistance manifested by CaCxSLCs.

Based on functional and/or phenotypic markers, identification and isolation of cancer stem-like cells in cervical cancer have been attempted by a number of investigators in recent years^5, 7, 8, 27, 28^. In contrast to these studies that employed single phenotypic/functional marker for isolation of CSC, we employed sequential gating and intermittent culturing of CaCxSLCs and enriched CaCxSLCs^6^. The process resulted in gradual enrichment of CSCs and was helpful in collecting higher number of CSCs for subsequent examination of their AP-1 response to UV exposure. Our data demonstrate a higher proportion of FTC-sensitive SP population in HPV-positive cells than the HPV-negative cells where the SP cells were barely detectable. Similar observations showing characteristically high proportion of FTC-sensitive CaCxSLC in HPV-positive cell lines have been reported recently^29^. We observed that HPV-positive SP cells effectively formed cervicospheres, a hallmark feature of stemness and self-renewal capacity^30^ but HPV-negative SP cells (C33a) failed to form cervicosphere of threshold size to be counted as spheres^6^. Similar low levels of SP fraction have been reported by other studies^9, 29, 31, 32^. Apart from ABCG2/SP-based sorting, after an intermittent culturing in low adhesion environment, we subjected these sorted cells to gating based on the epithelial stem cell markers CD49f and CD71^33^ and then on CD133^+ve^ phenotype^6^ that is associated with radioresistance^34^ for further isolation and enrichment of cervical CSCs. Application of triple gating demonstrated a gradual increase in sphere forming efficiency (SFE) in HPV-positive SP cells (SiHa) as compared to HPV-negative SP cells (C33a) that failed to form cervicosphere suggesting a potential utility of sequential gating with sorting and re-culturing of stem cells at each stage, to be a better strategy for enriching the cervical CSCs^6^. Serial passaging of cells in low adhesion cultures without gating have been attempted earlier but implementation of repeated use of single approach i.e. sphere formation resulted in only a mild increase in sphere forming ability^7^. However, in an improved protocol, we employed sequential gating and intermittent culturing that helps in enrichment of cervical CSC to a certain extent but further passaging with any other marker did not increase the SFE^6^. This may be due to the existence of an intrinsic inter-conversion and dynamic equilibrium that maintain the homoeostasis control of the cell subpopulations^35^.

Our earlier study on AP-1 in cervical cancer has demonstrated a constitutively active and overexpressed AP-1 in cervical cancer lesions and cell lines^10^. In the present investigation, we observed heterogeneity in expression/activation of AP-1 in cancer cells. An increased HPV-specific AP-1 DNA-binding activity was noted specifically in CaCxSLCs as compared to non-CaCxSLCs and it got further elevated when this cell population was subjected to UV-irradiation. This observation indicates differential response of the cancer cells to UV radiation based on their property of stemness. It was interesting to note that non-CaCxSLCs were not capable of sustaining their AP-1 activity post UV radiation and died. On the other hand, CaCxSLCs showed a sustained and/or enhanced AP-1 activity and enhanced proliferation. This differential response suggested capability of CaCxSLCs to maintain an active AP-1 signaling post radiation. Why this subset of cells show a differential response is currently not understood and needs further investigation. It is however, shown that JNK, an extracellular signal regulated protein kinase (ERK) plays an important role in UV-induced AP-1 activation and it appears to be the primary mechanism of radioresistance^15, 36^. Nevertheless, with two AP-1 specific binding sites in the URR of HPV16/18, AP-1 is among the strongest regulators of viral oncogene expression^37, 38^ and silencing oncogene expression in cervical cancer stem-like cells have been shown to inhibit their cell growth and self-renewability^6^. AP-1 has been independently shown to govern stemness in other cancers^39, 40^. Together, these observations suggest an important role of AP-1 in maintenance of stem cell pool in tumors and thus help in manifestation of properties associated with cancer stem cells such as radioresistance.

We observed an increased expression of AP-1 members in CaCxSLCs mainly c-Jun, c-Fos and JunB both at transcript as well as protein level but expression of Fra-1 that has been shown to be a tumor suppressor^10, 41^, remained completely absent both at transcript and protein level. On the other hand, in non-CaCxSLCs, the expression of most of the AP-1 members except JunB was undetectable at both transcript and protein level. We also observed nuclear localization of key members of AP-1 in CaCxSLCs indicating constitutively active involvement of AP-1 in CaCxSLCs. Interestingly, these differences in composition of AP-1 in the two cell populations were also reflected in their response to UV radiation in experimental setup. UV-treated CaCxSLCs showed increased expression of c-Fos and c-Jun whereas JunB and JunD showed decreased expression in UV exposure duration dependent manner as compared to non-CaCxSLCs which display complete loss of JunB. Similar observation showing characteristically higher level of c-Fos and c-Jun in stem cells has been reported earlier^40, 42, 43^. Studies also showed that control of c-Fos transcript and its protein levels are independently regulated. UV-irradiation was found to induce synthesis and post-translational modification of c-Fos and c-Jun proteins in irradiated epithelial cells^44^ whereas protein expression of c-Fos and c-Jun was found additionally negatively regulated by miR-101 and miR-155^45, 46^. Moreover, a recent report demonstrated that knockdown of AP-1 complex significantly sensitize prostate cancer cell to radiation^15^. Recently, our group showed that knockdown of Fra-2 sensitize chemo-radioresistant tongue tumor cells^18^. Similarly, RNAi-mediated knockdown of c-Jun sensitized radioresistant human nasopharyngeal carcinoma cells to radiation^20^. In addition, the change in AP-1 member expression resulted in increased cervicosphere size in CaCxSLC cultures and corresponding loss of cell viability in non-CaCxSLCs. These results indicate a pivotal role of c-Fos and c-Jun (AP-1) in imparting radioresistance in cervical cancer stem cells.

To validate functional role of AP-1, we targeted this transcription factor by a potent antioxidative herbal (polyphenolic) compound curcumin which showed a strong anti-AP-1 activity in cervical and oral cancer cells^10, 23^. Our result demonstrate that pre-treatment of CaCxSLC cultures with curcumin prior to UV exposure not only abrogated the basal AP-1 activity but also prevented post-exposure UV-induced increase in constitutively active AP-1 expression in these cells. Radiosensitizing role of curcumin has been demonstrated in different cancers^24, 25^, however, the mechanism(s) by which curcumin radiosensitizes the cancer cells remained elusive. It is quite possible that since curcumin strongly down regulates determinants of stemness, almost all upregulated gene, transcription factors and signalling pathways, it makes the cells sensitive to chemo-radiation making the treatment most effective.

Our study demonstrates that inhibition of AP-1 particularly in CSCs could be one of the key underlying mechanisms of radiosensitization by curcumin. This effect was accompanied by an enhanced anti-proliferative and pro-apoptotic effects while reducing expression of AP-1 members, c-Jun and c-Fos but significant upregulation of Fra-1. This is in concordance to our earlier observation in cervical cancer demonstrating similar expression dynamics of c-Fos and Fra-1 in cervical cancer cells following curcumin treatment^10^. The underlying mechanism(s) of this phenomenon are currently unknown; however, earlier study showed complete loss of DNA bound c-Jun/c-Fos complexes and resultant inhibition of AP-1 response genes by ectopically expressed Fra-1^47^ or by modulating gene expression via exerting epigenetic changes induced by curcumin in cancer cells^35, 48–50^ that also encompass members of AP-1 family^51^. Nevertheless, overexpression of Fra-1 is known to inhibit cell proliferation, induce apoptosis and reduce tumorigenicity in other cell types^52^. It has also been demonstrated that upregulation of Fra-1 leads to chemosensitization of breast cancer stem cells^53^. In contrast, a recent study shows Fra-1 downregulation in cervical cancer and it promotes apoptosis^54^. Further, Fra-1 through a positive feedback loop via miR-134 & SDS22 amplifies ERK and JNK signaling and reduce chemosensitivity in ovarian cancer cells^55^. We also observed an upregulation of Fra-1 [Fig. 5B(i), **Lane 3**]. However, action of Fra-1 was dependent on expression of other AP-1 members which were absent when the cells were treated with curcumin. The Fra-1 pool observed in curcumin/curcumin + UV treated cells could be an effect rather than cause of curcumin-induced loss of c-Jun and c-Fos. Similar accumulation of Fra-1 is observed by us *in vitro* and others in curcumin treated cervical and oral cancer cells^10, 18, 56, 57^. However, further studies are needed to establish role of Fra-1 in this phenomenon.

Loss of AP-1 activity was accompanied by loss of cells with stem-like properties in CaCxSLC cultures post-UV exposure. This inhibitory effect was also reflected on cervicospheres that were significantly eliminated. Intriguingly, curcumin have recently been shown to be effective in lowering tumor recurrence by targeting the CSC population, hence inhibiting tumor growth^58^. However, the mechanism by which it targeted cancer stem cells remained poorly understood. Studies addressing the effect of curcumin on resistance-linked ATP-binding cassette transporter membrane protein (ABCG2), a molecular determinant of stemness^59^, revealed specific inhibition by curcumin by binding to a site other than the FTC binding site^60^. This could be an additional means of targeting cancer stem cells apart from its inhibitory effects on AP-1 in CaCxSLCs. Furthermore, our combined *in vitro* and *in silico* data provide evidence for the inhibition of ABCG2 by curcumin supporting previous study in mice^60^.

Taken together our study showed for the first time existence of an increased AP-1 activity and preferential dose-dependent overexpression of c-Fos and c-Jun in cervical cancer stem-like cells in response to UV radiation indicates its role in radioresistance. Targeting AP-1 with a pharmacologically safe herbal derivative curcumin appears to induce radiosensitization of cancer stem cells through downregulation of c-Fos, c-Jun and upregulation of Fra-1 to make cancer treatment most effective.

## Materials and Methods

### Cell lines and cell culture

Human cervical cancer cell lines SiHa (HPV16 + ve)^61^ and C33a (HPV-ve)^62^ were obtained from the American Type Culture Collection (ATCC), USA. The cells were maintained using the DMEM (Sigma-Aldrich) medium containing 10% heat-inactivated fetal calf serum (Sigma-Aldrich) at 37  °C in a humidified atmosphere containing 5% CO_2_.

### SP analysis by flowcytometer using DCV labeling

Side population (SP) analysis was performed as described previously^63^. Briefly, 1 × 10^6^ cells from cervical cancer cell lines were incubated at 37 °C for 30 minutes with or without Fumitremorgin C (FTC; Alexis Biochemical) and then stained with dye cycle violet (DCV; 10 mmol/L; Life 124Technologies) for 90 minutes. The cells were then treated with 7-aminoactinomycin D (7-AAD; BD Biosciences) to discriminate viable cells. Data were collected on FACSAriaIII cell sorter (BD Biosciences) and analyzed using FlowJo (TreeStar). For SP and NSP gating, we ran the experiment in two parallel setup one with and other without 10 mmol/L FTC as described by Telford and colleagues^63^. In brief, cells were distinguished from debris on flowcytometer based on forward scatter (FSC) and side scatter (SSC). Doublets and aggregates were gated out based on SSC area (SSC-A) versus height (SSC-H) to ensure that a detected signal arises from single cells. Dead cells were recognized by their strong positivity for 7-AAD. The DCV fluorescence was excited with violet laser at 407 nm and was measured with 450/40BP (DCV-Blue) and 565LP (DCV-Red) filters and was displayed as dual fluorescence dot plot on a linear scale in presence or absence of FTC. Later, a gate drawn on the limit of DCV^dim^ staining during FTC inhibition included fewer SPs cells recognized as a dim tail extending from main population with a characteristic low fluorescence, whereas intense fluorescence signals of bulk population were defined as NSP cells (DCV^bright^). Finally, for sorting, DCV^dim^ (SP) and DCV^bright^ (NSP) cells in combination with fluorescent-labelled specific antibodies were analyzed for stem cell marker expression.

### Cervicosphere culture

Cervicosphere cultures were established as described by Dontu and colleagues with minor modifications^64^. In brief, 1 × 10^4^ cells/well were seeded on 6-well plates (Corning) precoated with 1.2% Poly-HEMA (Sigma-Aldrich) in defined conditioned medium (DCM) consisting of K-SFM (Invitrogen) supplemented with 10 ng/mL basic fibroblast growth factor (BD Biosciences), 10 ng/mL EGF (Sigma Aldrich) and B27 (Invitrogen). Sphere forming efficiency (SFE) was calculated using the procedure described earlier^65^. Subsequently, 2° and 3° cervicospheres were generated by culturing in 1.2% Poly-HEMA precoated 6-well plates.

### Flow cytometry analysis, sorting and isolation of CSCs and their enrichment

Flow cytometry analysis was done as described earlier^6^. Cells (1 × 10^5^) obtained after enzymatic dissociation of 1° and 2° cervicospheres were stained with anti-CD49f-FITC (GoH3; BD-Pharmingen), anti-CD71-APC (M-A712; BD-Pharmingen), and/or anti-CD133-PE for 60 minutes at 4 °C in staining buffer (2% BSA in 185 PBS). Corresponding isotypes were used as control. Cells were washed in PBS, centrifuged, and finally resuspended in 300 µL analysis buffer (1% BSA/2 mmol/L EDTA in PBS). FACS sorting was done as described earlier^6^. In brief, 1 × 10^6^ cells were incubated with anti-CD49f-FITC, anti-CD71-APC, and/or anti-CD133-PE, and were finally resuspended in 500 µL analysis buffer. The dead cells and debris were excluded after 7-AAD staining (Invitrogen). Data were collected on FACSAriaIII cell sorter (BD Biosciences) and analyzed using FlowJo software (TreeStar).

### Isolation of CaCxSLC cells

Cervical cancer stem cells were isolated from HPV-positive and HPV-negative cervical cancer cell lines by triple gating as described in Fig. 1A. In brief, the process included isolation of side population (SP) cells followed by culturing and regated on phenotypic markers CD49f and CD71 and again re-culturing and finally gating on CD133. The sorted cells were examined for their stemness property by cervicosphere formation assay using intermittent culturing in low adherence defined conditioned medium (DCM). Sorted cells subjected to sphere formation generated cervicospheres only in cultures seeded with SP → (CD49f^+ve^ CD71^−ve^) → CD133^+ve^ cells were designated as CaCxSLCs whereas monolayers generated from non-side population (NSP) NSP → (CD49f^−ve^ CD71^+ve^) → CD133^−ve^ cells cultured in complete media (CM) in low adherence state were designated as non-CaCxSLCs. Same strategy has been used in each experiment.

### Confocal imaging and analysis

Staining of about 10–20 cervicospheres or parental cells was done in uncoated chamber slide (Corning) as described by Weiswald^66^. A Leica TCS SP5 confocal microscope was used to view the immunoflourescence. The 488, 594 or 633 nm laser lines were used for excitation of the fluorophores, while emissions were collected by specific band pass filters. DAPI as nuclear stain and Fluoromount as an antifade agent were used.

### Quantitative real-time PCR (qPCR)

Total cellular RNA was isolated from all cell population using TRI reagent according to the manufacturer’s protocol (Sigma Chemicals, USA). The quality and integrity of extracted RNA was checked spectrophotometrically and on 1.0% agarose gel. For Quantitative real-time PCR (qPCR), 3 μg of total RNA was used to prepare cDNA using the Fermentas First Strand cDNA Synthesis kit (Thermo Scientific, USA) according to manufacturer’s protocol and was performed as previously described using a Bio-Rad iCycler^6^. All quantifications were normalized to the level of GAPDH transcripts which was used as input control. Primer sets are listed in Supplementary Table 1.

### Immunoblotting

Immunoblot analysis was performed for different cell phenotypes by the method described previously^67^. Total cellular proteins (50 μg/lane) were separated on 12% polyacrylamide gel and electro-transferred on PVDF membranes (Millipore Corp, USA). The membrane was blocked in PBS containing 5% non-fat skimmed milk and probed with specific antibodies against cFos (H-125), Fra-1 (R-20), cJun (N), JunB (210) and JunD (329) by incubating the membrane overnight in pre-standardized dilution of primary antibody in blocking solution at 4 °C. These blots were washed, incubated with HRP-anti-mouse IgG secondary antibodies and visualized by Luminol detection kit (Santa Cruz Biotech) by exposing the blot to KODAK X-Omat films (Kodak India, India). The Western blot membranes were stripped and reprobed for β-actin (C-11) expression which was used as an internal control. The quantitative densitometric analysis of the bands was performed using Alpha Ease FC version 4.1.0 (Alpha Innotech Corporation, IL).

### Electrophoretic Mobility Shift Assay (EMSA)

EMSA was performed as described previously^67^. Briefly, 10 μg of nuclear extract was incubated with γ-^32^P-radiolabeled AP-1 of HPV-16 5′-ATAAAGGTTAGTCATACATTGTTC-3′; positions 7804 to 7827-3′^68^ for 30 min in 25 μl of reaction buffer. For the competition assay, 100x molar excess of unlabeled oligo (AP-1) and non-specific oligo (Oct-1-5′-TGTCGAATGCAAATCACTAGAA-3′) was added. Protein–DNA complexes were resolved in 4.5% nondenaturing polyacrylamide gel (crosslinking ratio, 29:1) and exposed to phosphorimager (Fujifilm FLA-5100) using MultiGauge-ver 3.x analysis software. The quantitative densitometric analysis was performed using Alpha Ease FC version 4.1.0 (Alpha Innotech Corporation, IL).

### UV irradiation

Briefly, cells with different phenotype were rinsed twice with PBS and were treated with either 50 J/m^2^ or 100 J/m^2^ UV (254 nm) by CL-1000 Ultraviolet Crosslinker equipped with UV lamps (Upland, CA, USA) as per the required experiment. The retained medium was added back to the cells and analyses were performed at different lengths of time post-irradiation as indicated in the figure legends. Unsorted untreated parental SiHa cells were used as control.

### Apoptotic cell death analysis by Annexin V-FITC and AO/EtBr staining

The extent of apoptosis in CaCxSLCs cells was evaluated by flow cytometric analysis using FITC-conjugated Annexin-V/Propidium iodide kit (BD Pharmingen) as described previously^12^. Total apoptotic cell death was determined by early apoptotic and late apoptotic cells. Further confirmation of apoptotic cell death was done by acridine orange (50 µg/ml, Sigma Aldrich) and ethidium bromide (1 µg/ml, Sigma Aldrich) staining as per the manufacturer’s instruction.

### Cell cycle analysis

Cell cycle analysis was done using flow cytometry. Cells (1 × 10^5^) were fixed in ice cold 70% ethanol, incubated overnight at −20 °C and stained with PI/RNAse solution (BD Bioscience) for 15 min at 37 °C. Cell cycle analysis was performed using FACSAriaIII cell sorter and cell percentages in each phase of the cell cycle were analysed using FlowJo software.

### *In vivo* tumor inhibition assay

All animal experiments were approved by Institutional Animal Ethics Committee of INMAS (DRDO) and ACBR, University of Delhi, Delhi and the procedure was carried out in accordance to CPCSEA guidelines (Committee for the Purpose of Control and Supervision on Experiments on Animals). Female athymic nude mice at 8–10 weeks of age were made into four groups as described in Supplementary Table 2. The tumor sizes were measured using vernier callipers biweekly and tumor volume (mm^3^) was calculated using the standard formula: (LxW^2^)/2. Each mouse were euthanized when the tumors diameter reached ~1.0 cm and were either fixed in 4% neutral, buffered formaldehyde for histological assessment or collected in Trizol or PBS for molecular analysis.

### Immunohistochemical analysis

Immunocytochemistry on cell populations and immunohistochemistry on formalin-fixed paraffin embedded tissue sections were performed as described by Janzen and colleagues^69^ with indicated antibodies and were imaged on an Olympus IX81 upright microscope equipped with cooled CCD camera and Image Pro-Plus software (Media Cybernetics). For histopathological analysis, tissue sections (5 μm) were stained with hematoxylin and eosin.

### *In Silico* docking analysis

The three dimensional crystal structure of ABCG2 was obtained from Protein Data Bank (PDB ID: 4A82)^70^. Curcumin structure was retrieved from NCBI PubChem Compound database (PubChem Compound ID: 969516). For molecular docking simulation, structures were submitted to FireDock server and the docked poses were visulalized using Pymol^71^.

### Statistical analysis

The data analyses were performed using the statistical software GraphPad Prism (version 5.0) (GraphPad Software, Inc., California). All cell culture experiments were carried out at least in three independent experimental runs. Statistical significance of difference between the two test groups was analyzed by the Student’s t-test and multiple comparisons versus control group were assessed by one-way ANOVA with post-hoc Tukey’s ANOVA. *p-*values < 0.05 were considered statistically significant.

## Electronic supplementary material

Supplementary File
