# Supplementary material for: Cervical cancer stem cells manifest radioresistance: Association with upregulated AP-1 activity
Source: Sci Rep. 2017 Jul 6;7:4781. doi: 10.1038/s41598-017-05162-x (PMC5500478; doi:10.1038/s41598-017-05162-x)
Supplement: Supplementary file 1 — Supplementary File [file 41598_2017_5162_MOESM1_ESM.pdf]

Supplementary Section

Cervical cancer stem cells manifest radioresistance: Association with upregulated AP-1 activity

Abhishek Tyagi[1,2,3], Kanchan Vishnoi[2], Harsimrut Kaur[1], Yogesh Srivastava[2], Bal Gangadhar Roy[4], Bhudev C. Das[1,3*] and Alok C. Bharti[2,5*]

1 Molecular Oncology Laboratory, B.R. Ambedkar Centre for Biomedical Research (ACBR), University of Delhi, Delhi, 110007, India
2 Division of Molecular Oncology, Institute of Cytology & Preventive Oncology (ICMR), Noida, 201301, Uttar Pradesh, India
3 Stem Cell and Cancer Research Lab, Amity Institute of Molecular Medicine and Stem Cell Research (AIMMSCR), Amity University, Uttar Pradesh, Noida, 201313, India
4 Institute of Nuclear Medicine and Allied Sciences, Defence Research Development Organization, Delhi, 110 054, India
5 Molecular Oncology Laboratory, Department of Zoology, University of Delhi, Delhi, 110007, India

***Corresponding Authors:**

Prof. Alok C. Bharti,
Molecular Oncology Laboratory, Department of Zoology,
University of Delhi, Delhi – 110007, India
Tel: +91-8800171246
E-Mail:-alokchandrab@yahoo.com

Prof. Bhudev C. Das,
Amity Institute of Molecular Medicine & Stem Cell Research (AIMMSCR),
Amity University, Sector-125, Noida, 201313, Uttar Pradesh, India.
Tel: +91-120-2586855, +91-9810566870
Fax: +91-120-4392114
E-Mail:- bcdas@amity.edu

Supplementary Table 1: List of real-time RT-PCR primers used in the study

S.No.	Primers	Sequence	References
**1.**	**c-Fos**	F; 5’-CGGGTTTCAACGCCGACTA-3’<br>R; 5’- TTGGCACTAGAGACGGACAGA-3’	1
**2.**	**Fra-1**	F; 5’- CCCTGCCGCCCTGTACCTTGTATC-3’<br>R; 5’-GACATTGGCTAGGGTGGCATCTGCA-3’	2
**3.**	**c-Jun**	F; 5’-AGAGCGGTGCCTACGGCTACAGTAA -3’<br>R; 5’-CGACGTGAGAAGGTCCGAGTTCTTG -3’	3
**4.**	**JunB**	F; 5’-TCTCTCAAGCTCGCCTCTTC-3’<br>R; 5’-ACGTGGTTCATCTTGTGCAG-3’	2
**5.**	**JunD**	F; 5’-TTCTACTCGGGGAACAAACG-3’<br>R; 5’-GGCGAACCAAGGATTACAAA-3’	2

Supplementary Table 2: Inhibition of tumor growth in athymic nude mice

Treatment*	No. of Mice (n=24)	Mice with Tumor**	Tumor size (cm^3)
**Vehicle (DMSO)**	6	5 (100%)	1.2 (±0.01)
**Cur(25µM)**	6	3 (50%)	0.26 (±0.08)
**UV(50J/m^2)**	6	6 (80%)	2.7 (±0.2)
**Cur(25µM)+ UV(50J/m^2)**	6	0 (0%)	-

*CaCxSLCs ($20x10^3$) were s.c. injected for obtaining xenograft tumor.
** Tumor formations after 4-weeks post injection in athymic nude mice.

References:

1 Schulze, J. *et al.* Fos-dependent induction of Chk1 protects osteoblasts from replication stress. *Cell Cycle* **13**, 1980-1986, doi:10.4161/cc.28923 (2014).

2 Macleod, K. *et al.* Altered ErbB receptor signaling and gene expression in cisplatin-resistant ovarian cancer. *Cancer Res* **65**, 6789-6800, doi:65/15/6789 [pii]10.1158/0008-5472.CAN-04-2684 (2005).

3 Jiao, X. *et al.* c-Jun induces mammary epithelial cellular invasion and breast cancer stem cell expansion. *J Biol Chem* **285**, 8218-8226, doi:10.1074/jbc.M110.100792 (2010).

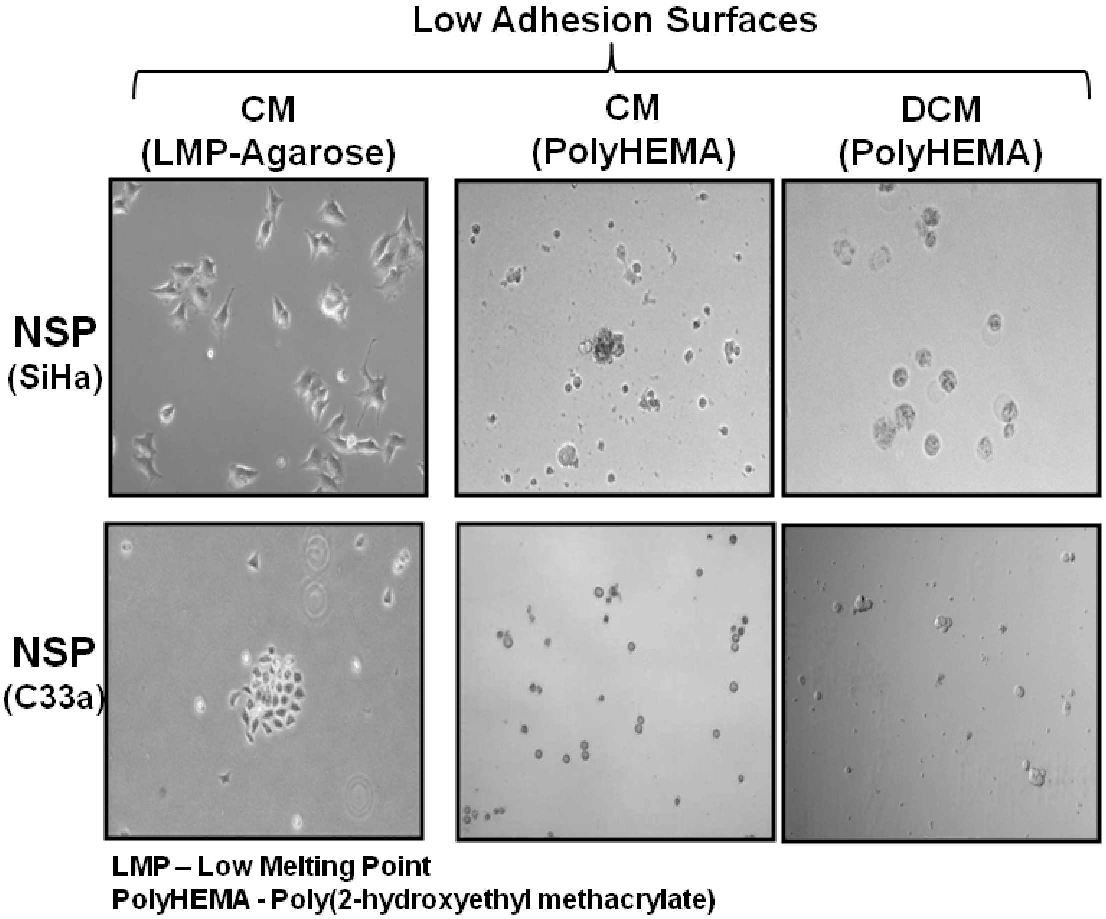

Supplementary Figure S1: Culturing of NSP cells on low adhesion surfaces. Sorted NSP cells from SiHa and C33a cells were cultured in comparable low adhesion surfaces (polyHEMA and LMP-agarose) and media conditions [defined conditioned media (DCM) or complete media (CM)] for assessment of cervicosphere formaing ability.
